# Near Infra-Red Photoimmunotherapy with Anti-CEA-IR700 Results in Extensive Tumor Lysis and a Significant Decrease in Tumor Burden in Orthotopic Mouse Models of Pancreatic Cancer

**DOI:** 10.1371/journal.pone.0121989

**Published:** 2015-03-23

**Authors:** Ali A. Maawy, Yukihiko Hiroshima, Yong Zhang, Roger Heim, Lew Makings, Miguel Garcia-Guzman, George A. Luiken, Hisataka Kobayashi, Robert M. Hoffman, Michael Bouvet

**Affiliations:** 1 Department of Surgery, University of California San Diego, San Diego, California, United States of America; 2 AntiCancer, Inc., San Diego, California, United States of America; 3 Department of Surgery, Yokohama City University, Yokohama City, Japan; 4 Aspyrian Therapeutics, San Diego, California, United States of America; 5 OncoFluor, Inc., San Diego, California, United States of America; 6 National Institutes of Health, Bethesda, Maryland, United States of America; 7 Surgical Service, VA Healthcare System, San Diego, California, United States of America; University of Nebraska Medical Center, UNITED STATES

## Abstract

Photoimmunotherapy (PIT) of cancer utilizes tumor-specific monoclonal antibodies conjugated to a photosensitizer phthalocyanine dye IR700 which becomes cytotoxic upon irradiation with near infrared light. In this study, we aimed to evaluate the efficacy of PIT on human pancreatic cancer cells in vitro and in vivo in an orthotopic nude mouse model. The binding capacity of anti-CEA antibody to BxPC-3 human pancreatic cancer cells was determined by FACS analysis. An in vitro cytotoxicity assay was used to determine cell death following treatment with PIT. For in vivo determination of PIT efficacy, nude mice were orthotopically implanted with BxPC-3 pancreatic tumors expressing green fluorescent protein (GFP). After tumor engraftment, the mice were divided into two groups: (1) treatment with anti-CEA-IR700 + 690 nm laser and (2) treatment with 690 nm laser only. Anti-CEA-IR700 (100 μg) was administered to group (1) via tail vein injection 24 hours prior to therapy. Tumors were then surgically exposed and treated with phototherapy at an intensity of 150 mW/cm^2^ for 30 minutes. Whole body imaging was done subsequently for 5 weeks using an OV-100 small animal imaging system. Anti-CEA-IR700 antibody bound to the BxPC3 cells to a high degree as shown by FACS analysis. Anti-CEA-IR700 caused extensive cancer cell killing after light activation compared to control cells in cytotoxicity assays. In the orthotopic models of pancreatic cancer, the anti-CEA-IR700 group had significantly smaller tumors than the control after 5 weeks (p<0.001). There was no significant difference in the body weights of mice in the anti-CEA-IR700 and control groups indicating that PIT was well tolerated by the mice.

## Introduction

Photoimmunotherapy (PIT) uses tumor specific monoclonal antibodies that are conjugated to the photosensitizer phthalocyanine dye, IR700, which is cytotoxic upon irradiation with near-infrared (NIR) light [1–3].

Several monoclonal antibodies (mAbs) have been used with PIT in mouse models of breast cancer including trastuzumab, a monoclonal antibody (mAb) directed against human epidermal growth factor receptor-2 (HER-2), and panitumumab, a monoclonal antibody directed against human epidermal growth factor receptor-1 (HER-1) [4,5]. Cell death was induced immediately after irradiating mAb-IR700–bound target cells with NIR light. In vivo tumor shrinkage after irradiation with NIR light was demonstrated in target cells expressing the epidermal growth factor receptor. The mAb-IR700 conjugates were effective when bound to the cell membrane and produced no phototoxicity when not bound, suggesting a different mechanism for PIT as compared to conventional photodynamic therapies [1].

Pancreatic cancer is a lethal tumor with high rates of local and distant recurrence [6,7]. In the present study, we used a chimeric monoclonal antibody against the carcinoembryonic antigen (CEA) for PIT, which is often overexpressed in pancreatic cancer and has been previously utilized by our laboratory for fluorescence-guided surgery and fluorescence laparoscopy [8–17]. The anti-CEA antibody was conjugated to IR700 and used for PIT treatment of human pancreatic cancer in orthotopic mouse models as well as pancreatic cancer cells in vitro.

## Materials and Methods

### Cell Culture

The human pancreatic cancer cell line BxPC-3 was stably transduced to express green fluorescent protein (GFP) as previously described [18,19]. Cells were maintained in RPMI 1640 medium supplemented with 10% fetal bovine serum (Hyclone, Logan, UT), penicillin/streptomycin (Gibco-BRL, Carlsbad, CA), sodium pyruvate (Gibco-BRL), sodium bicarbonate (Cellgro, Manassas, VA), l-glutamine (Gibco-BRL), and minimal essential medium nonessential amino acids (Gibco-BRL). All cells were cultured at 37°C in a 5% CO_2_ incubator.

### Determination of CEA antigen expression level

BxPC-3-GFP cells from a 75 cm^2^ flask were harvested with enzyme-free cell dissociation buffer, washed once with incubation buffer (PBS + 0.5% FBS + 0.1% sodium azide), and recovered in incubation buffer at 2 x 10^6^ cells/ml and kept at 4°C. The cells (5 x 10^5^) were incubated with chimeric anti-CEA antibody (Genara Biosciences LLC, Morgan Hill, CA) (10 μg/ml) in 300 μl incubation buffer for 1 hour at 4°C, washed three times with PBS and stained with an Alexa488-conjugated donkey anti human IgG (H+L) antibody (Jackson Immunoresearch, West Grove, PA) for 45 minutes, followed by three washes with PBS. A control without anti-CEA was prepared in parallel. Flow cytometry profiles from the anti-CEA antibody-treated cells and the untreated cells were established on a Guava EasyCyte Plus flow cytometer (EMD Millipore, Billerica, MA). The antibody binding capacity for anti-CEA was determined by using reference beads (Bangs Laboratories, Inc., Fishers, IN) and geometric means according to the manufacturer’s protocol.

### Animals

Athymic nu/nu nude mice (AntiCancer Inc., San Diego, CA), 4–6 weeks old, were used in this study. Mice were kept in a barrier facility under HEPA filtration. Mice were fed with an autoclaved laboratory rodent diet. All mouse surgical procedures and imaging were performed with the animals anesthetized by intramuscular injection of 50% ketamine, 38% xylazine, and 12% acepromazine maleate (0.02 ml). Animals received buprenorphine (0.10 mg/kg ip) immediately prior to surgery and once a day over the next 3 days to ameliorate pain. CO_2_ inhalation was used for euthanasia of all animals at 5 weeks after surgery. To ensure death following CO_2_ asphyxiation, cervical dislocation was performed. All animal studies were conducted with an AntiCancer, Inc. Institutional Animal Care and Use Committee (IACUC)-protocol specifically approved for this study and in accordance with the principals and procedures outlined in the National Institute of Health Guide for the Care and Use of Animals under Assurance Number A3873–1.

### Antibody-Dye Conjugation

A water-soluble silicon-phthalocyanine derivative, IRDye 700DX NHS ester was obtained from LI-COR Bioscience (Lincoln, NE). of Chimeric anti-CEA antibody (Genara Biosciences LLC) (2 mg [~ 14 nmol]) at a concentration of 2 mg/ml in 0.1 M Na_2_HPO_4_ (pH = 8.6) was incubated for 2 hours at room temperature with IR700dye NHS ester (135 ug, 70 nmol) prepared in anhydrous DMSO at 5 mmol/L. After the incubation period, the IR700-conjugate was buffer exchanged and purified with phosphate buffer saline (PBS, pH = 7.1) using Amicon Ultra Centrifugal Filter Units (EMD Millipore Corporation, Billerica, MA). The IR700-mAb conjugate was repeatedly diluted with 10 ml volumes of PBS and then concentrated using the filter units until less than 2% of the unconjugated IR700 dye species remained, as determined by size exclusion HPLC (SE-HPLC). Analysis of the conjugates by SE-HPLC was performed using an Agilent 1100 HPLC system fitted with a TSKgel G2000SWxl column (Tosoh Biosciences, Tokyo, Japan). The SE-HPLC elution buffer was 1X PBS (pH = 7.1) with a flow rate of 1 ml/min. UV/Vis detection at 280 nm and 690 nm was used to determine the average dye-to-antibody ratio (DAR) for each conjugates. With this sample, a purity of 97.6% with 0.5% free dye and a DAR of 4.1 was achieved.

### Tumor implantation

After confluence, BxPC-3-GFP human pancreatic cancer cells (1 x 10^6^) were injected subcutaneously into the flanks of nude mice and allowed to engraft and grow over a period of 4–6 weeks. Tumors were then harvested and tumor fragments (1 mm^3^) from subcutaneous tumors were sutured to the tail of the pancreas using 8–0 nylon surgical sutures (Ethilon; Ethicon Inc., Somerville, NJ). On completion, the tail of the pancreas was returned to the abdomen, and the incision was closed in one layer using 6–0 nylon surgical sutures (Ethilon) [20,21]. The tumor fragments were allowed to grow over a period of 2 weeks.

### Cytotoxicity studies

BxPC-3 cells were seeded in white-wall 96-well plates (4,000/well) and allowed to attach overnight. Cells were incubated with the antibody conjugate, anti-CEA-IR700, (dye-antibody ratio of 5.1) at 10 mg/ml for 2 hours at 37°C. Four wells at a time were subjected to treatment with 690 nm light from an LED (Marubeni Corporation, Tokyo, Japan) at a power density of 50 mW/cm^2^ that was calibrated with a power meter equipped with a photodiode power sensor (Thorlabs Inc., Newton, NJ). After light treatment, the antibody solution was replaced with complete RPMI 1640 medium, containing CytoTox Green (Promega, Madison, WI) to monitor cell killing.

### Photoimmunotherapy in vivo

Anti-CEA antibody (100μg) (Genara Biosciences LLC, Morgan Hill, CA) conjugated to IR700DX reconstituted to 100 μl was injected via tail vein in the treatment group 24 hours prior to intervention, while the control group had 100 μl of PBS similarly injected 24 hours prior to injection. Each group consisted of 10 mice with orthotopic BxPC-3-GFP tumors.

After 24 hours, the pancreatic tumors in all 10 mice in the treatment group were exposed via a left lateral incision and imaged to detect both the GFP signal and the 700 nm signal. All the mice were subsequently subjected to photoimmunotherapy by exposing the tumor to a 690 nm laser at 150 mW/cm2 for 30 minutes for a total of 270 J/cm2. The surrounding normal tissues were protected with aluminum foil during PIT. Mice were imaged at the time of therapy and weekly thereafter with tumor exposed to evaluate response to therapy. After 5 weeks the mice were sacrificed, at which point they were imaged and had their tumors resected and weighed.

### Animal Imaging

Mice were imaged weekly using the Olympus OV100 small animal imaging system (Olympus Corp. Tokyo, Japan), containing an MT-20 light source (Olympus Biosystems Planegg, Germany) and DP70 CCD camera (Olympus Corp. Tokyo, Japan) [22]. All images were analyzed using Image-J (National Institute of Health Bethesda, MD) and were processed with the use of Photoshop elements-11 (Adobe Systems Inc. San Jose, CA).

### Tumor size determination

The mice in both groups had weekly laparotomy to expose the pancreatic tumors via a left lateral incision. Tumors were imaged with the OV-100 by GFP expression. Tumor size was assessed using Image-J software (National Institutes of Health, Bethesda, Maryland).

### Statistical Analysis

All statistical analysis was done using SPSS software version 21 (IBM, Armonk, NY). For pairwise comparisons, quantitative variables were calculated using the paired-samples Student’s t-test and confirmed with the Wilcoxon rank-sum test. A p-value ≤0.05 was considered significant. 95% confidence intervals obtained on analysis of the data were configured into the error bars of the appropriate figures and graphs.

## Results and Discussion

### Anti-CEA-IR700 binds to CEA-expressing pancreatic cancer cells in vitro and causes extensive cell death after light activation compared to control

**Fig 1:**
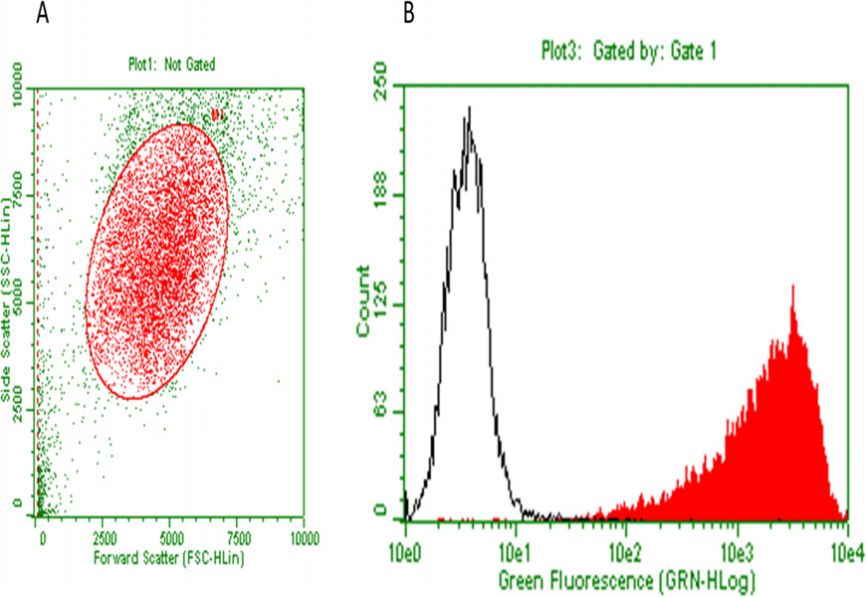
Staining of BxPC-3 cells with anti-CEA antibody and quantification of binding sites. BxPC-3 cells were treated with anti-CEA antibody and stained with a fluorochrome conjugated secondary antibody against anti-CEA (red, filled), or control-stained with secondary antibody only (black, open) and analyzed by flow cytometry. Gating was for the main population (A). The anti-CEA antibody binding capacity, determined with reference beads, was very high (2,227,000 binding sites per cell) (B).

**Fig 2:**
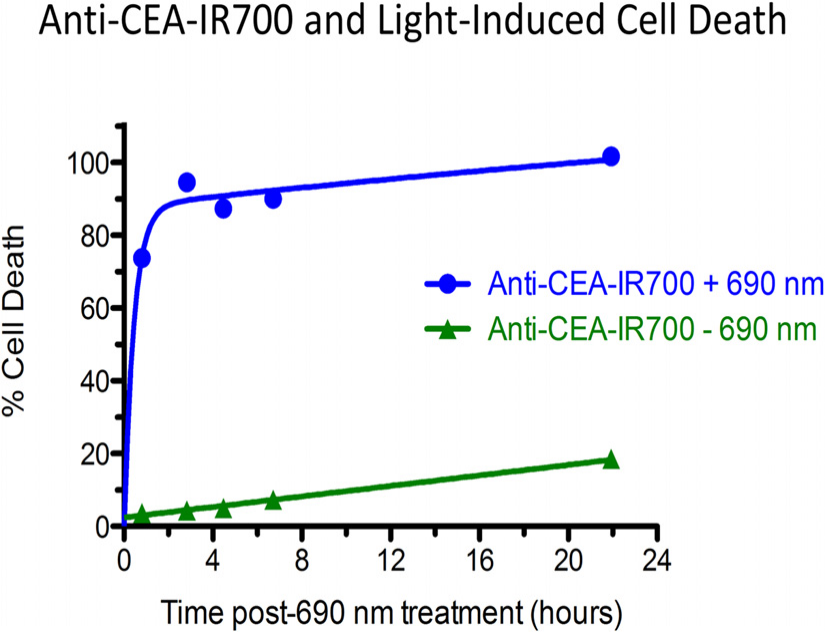
Light-induced cell death by cell-bound anti-CEA-IR700. BxPC-3 cells were incubated with anti-CEA-IR700 conjugate (10 μg/ml). Rapid cell killing was observed with a dose of 32 J/cm^2^ of 690 nm near-infrared light (blue circles), whereas only little cell killing occurred without illumination (green triangles). Death in light-treated cells in the absence of anti-CEA-IR700 was negligible (data not shown).

The anti-CEA antibody binding capacity to BxPC3 human pancreatic cancer cells was 2,227,000 binding sites per cell by FACS analysis (Fig. 1). Cells were incubated with anti-CEA-IR700 and treated with 690 nm light. At the end of the incubation, there was 100% cell death in the anti-CEA IR700 + 690 nm light group compared to a negligible amount of cell death in the no-690 nm light group (Fig. 2). Death of 690 nm light-treated cells in absence of Anti-CEA-IR700 was negligible (data not shown).

### PIT results in a significant reduction in tumor size and weight in an orthotopic mouse model of pancreatic cancer

**Fig 3:**
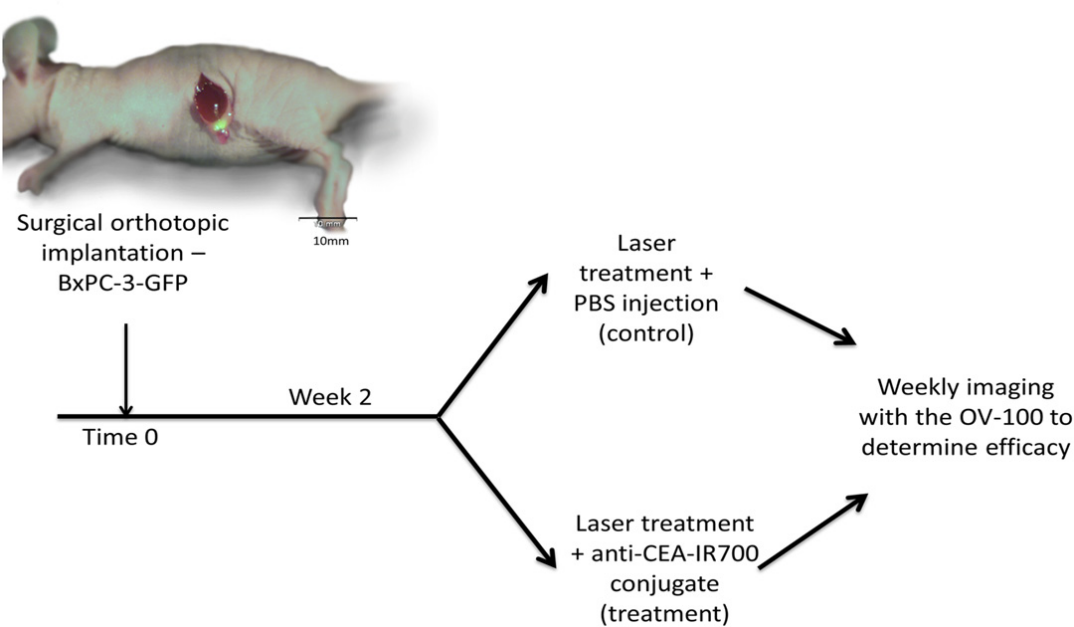
Experimental protocol for in vivo PIT. Two weeks after orthotopic implantation of BxPC-3-GFP tumors, engraftment was ensured and mice were divided into 2 groups with the treatment group receiving anti-CEA-IR700 conjugate (100 μg) and the 2^nd^ group receiving an injection with PBS. Mice were serially imaged weekly over the course of 5 weeks to measure orthotopic tumor growth.

**Fig 4:**
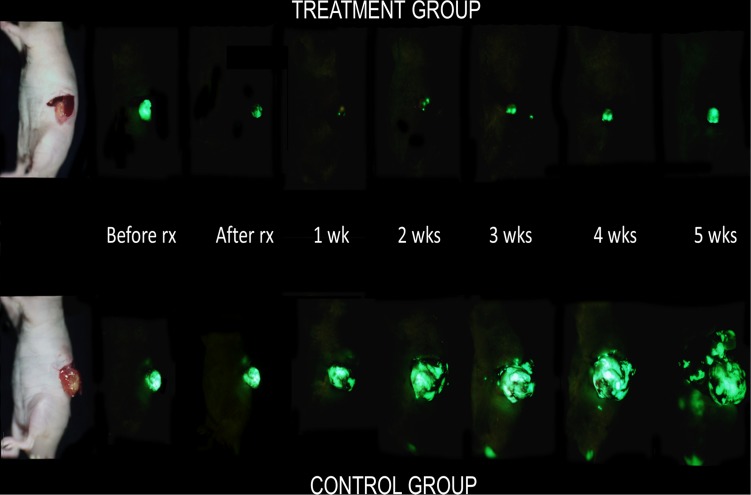
GFP weekly imaging of pancreatic tumors. Mice had their tumors exposed via a left lateral incision and imaged with the OV-100. Maximal effect of PIT was noted one week after initiation of therapy.

**Fig 5:**
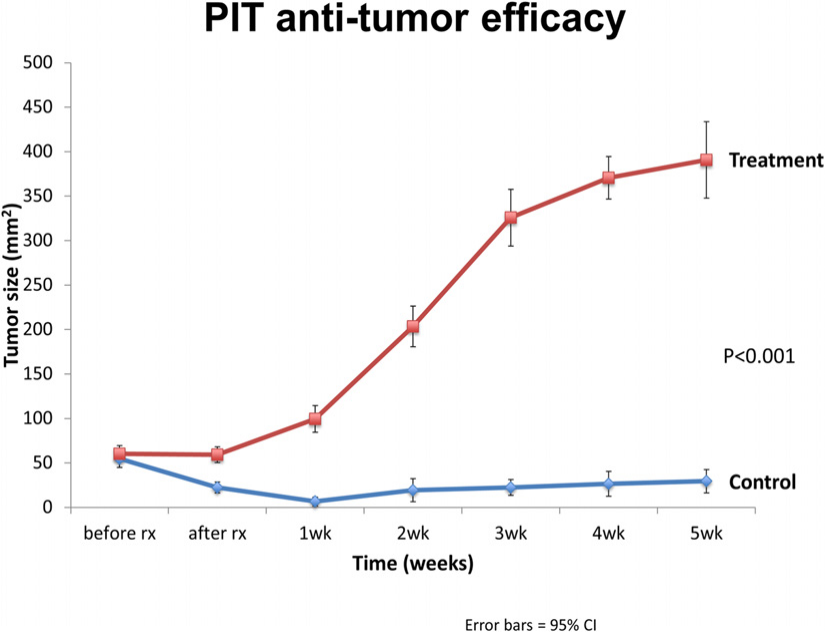
Graphical representation of tumor size over 5 weeks. Tumor sizes were determined using image-J software (National Institutes of Health, Bethesda, Maryland).

Two weeks after orthotopic implantation of BxPC-3-GFP tumors, engraftment was ensured and mice were divided into 2 groups with the treatment group receiving anti-CEA-IR700 conjugate (100 μg) and the control group receiving PBS (Fig. 3). Tumor size was assessed on a weekly basis to evaluate response to therapy and overall differences in progression. In the control group there was an initial exponential increase in tumor size that began to plateau at week 4 achieving a maximum average of 390.7 mm^2^ (95% CI [347.7, 433.7]). In contrast, in the treatment group there was an initial decrease in tumor size from baseline with a maximal response seen at week 1 (Figs. 4 and 5) with an average size of 6.65mm^2^ (95% CI [1.75, 11.5]). Over the course of the experiment, the tumor began to regrow, reaching a maximum average of 29.5 mm^2^ (95% CI [16.5, 42.5]) at 5 weeks post-treatment. The difference in tumor size between the control and the treatment groups was significant (p<0.001).

**Fig 6:**
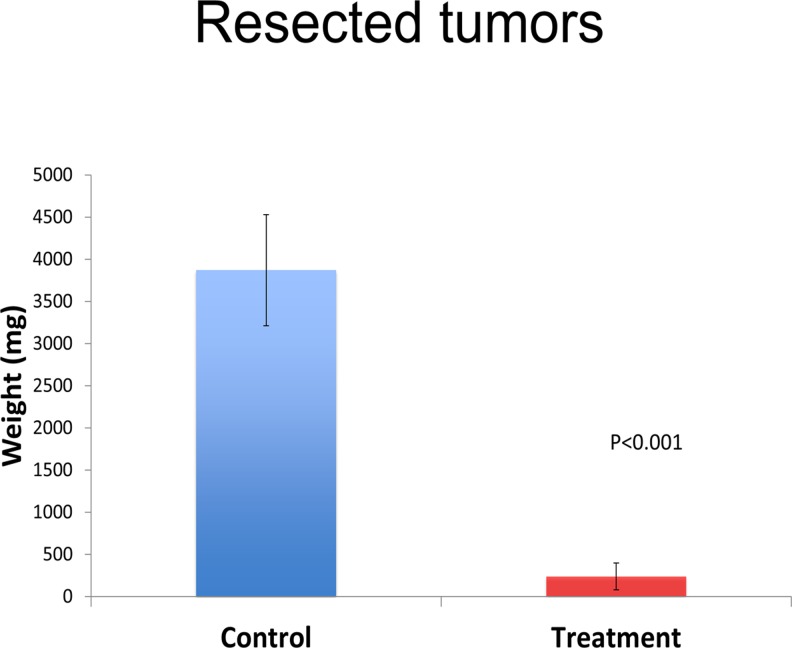
Graphical representation of tumor weights. A significant difference was noted with average tumor weights after tumor resection. A significant difference in tumor weight was noted between the treatment and control groups (p<0.001).

**Fig 7:**
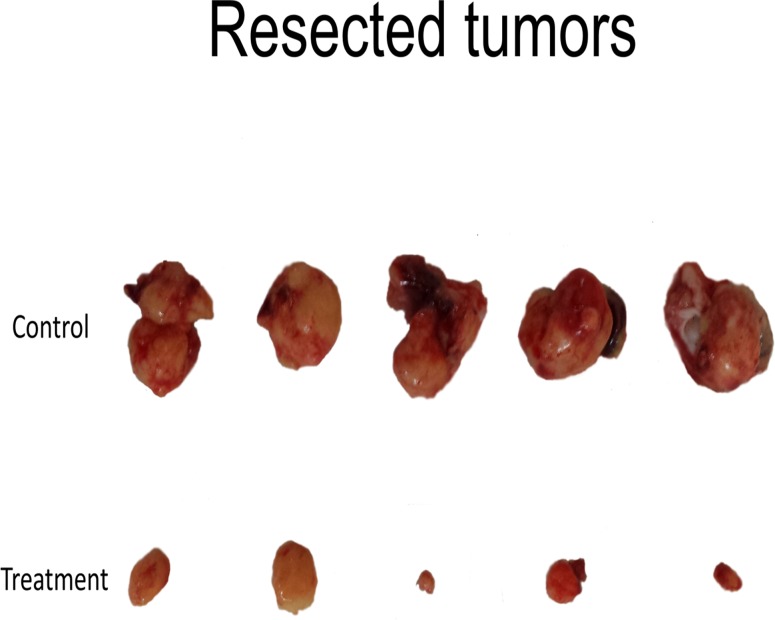
Resected tumors. The top panel shows the tumors from the control group and the bottom panel shows tumors from the treatment group.

At the termination of the experiment at week 5, the tumors in both groups were excised and weighed. Complete excision was confirmed with the OV-100 by GFP fluorescence. The average tumor weight was 3872 mg (95% CI [3213, 4531]) for the control group and 239.6 mg (95% CI [81, 397]) for the treatment group (p<0.001) (Figs. 6 and 7).

The average body weights of the mice after one week of treatment were 26.3 grams (95% CI [25.1, 27.4]) for the PIT group and 25.1 grams for the control group (95% CI [24, 26.2]) which was not statistically different (p = 0.23). The average body weights of the mice 5 weeks after treatment were 29.2 grams (95% CI [28, 30.5]) for the PIT group and 28.7 grams (95% CI [27, 30.3]) for the control group which was also not statistically different (p = 0.64), indicating that PIT was well tolerated by the mice.

Despite the anti-tumor effects of PIT, there was however a 100% recurrence rate. Previous studies have shown that the rate and amount of tumor cell destruction is dependent on both the conjugate dose and the light dose, the product of which results in the same cytotoxic effect regardless of the method of light delivery (continuous or intermittent) [2,23]. In this regard, further investigation is needed to assess how dosing of the anti-CEA-IR700 complex and varying the mode and amount of energy delivery could increase efficacy.

A single round of treatment was employed in the present report as proof of principle in an orthotopic model of pancreatic cancer. Multiple rounds of PIT will be performed in future experiments. Repeated rounds of therapy have been shown to increase the efficacy of PIT [4]. It is expected that repeated rounds of PIT would reduce the recurrence rate. Repeated rounds of PIT should be feasible due to the low toxicity observed. PIT should be able to add the efficacy of surgery and radiation therapy when used in combination with these therapies. This will be tested in orthotopic models, including immunocompetent mice, and in experimental high metastatic models, as well as in patient-derived orthotopic xenograft (PDOX) models in future experiments.

## References

[1] Mitsunaga M, Ogawa M, Kosaka N, Rosenblum LT, Choyke PL, Kobayashi H. Cancer cell-selective in vivo near infrared photoimmunotherapy targeting specific membrane molecules. Nat Med. 2011;17: 1685–1691. 10.1038/nm.2554 22057348 PMC3233641

[2] Nakajima T, Sato K, Hanaoka H, Watanabe R, Harada T, Choyke PL, et al The effects of conjugate and light dose on photo-immunotherapy induced cytotoxicity. BMC Cancer. 2014;14: 389 10.1186/1471-2407-14-389 24885589 PMC4055275

[3] Sato K, Watanabe R, Hanaoka H, Harada T, Nakajima T, Kim I, et al Photoimmunotherapy: comparative effectiveness of two monoclonal antibodies targeting the epidermal growth factor receptor. Mol Oncol. 2014;8: 620–632. 10.1016/j.molonc.2014.01.006 24508062 PMC4004687

[4] Mitsunaga M, Nakajima T, Sano K, Choyke PL, Kobayashi H. Near-infrared theranostic photoimmunotherapy (PIT): repeated exposure of light enhances the effect of immunoconjugate. Bioconjug Chem. 2012;23: 604–609. 10.1021/bc200648m 22369484 PMC3401044

[5] Mitsunaga M, Nakajima T, Sano K, Kramer-Marek G, Choyke PL, Kobayashi H. Immediate in vivo target-specific cancer cell death after near infrared photoimmunotherapy. BMC Cancer. 2012;12: 345 10.1186/1471-2407-12-345 22873679 PMC3502522

[6] Bouvet M, Gamagami RA, Gilpin EA, Romeo O, Sasson A, Easter DW, et al Factors influencing survival after resection for periampullary neoplasms. Am J Surg. 2000;180: 13–17. 11036132 10.1016/s0002-9610(00)00405-0

[7] Ryan DP, Hong TS, Bardeesy N. Pancreatic adenocarcinoma. N Engl J Med. 2014;371: 1039–1049. 10.1056/NEJMra1404198 25207767

[8] Hiroshima Y, Maawy A, Metildi CA, Zhang Y, Uehara F, Miwa S, et al Successful fluorescence-guided surgery on human colon cancer patient-derived orthotopic xenograft mouse models using a fluorophore-conjugated anti-CEA antibody and a portable imaging system. J Laparoendosc Adv Surg Tech A. 2014;24: 241–247. 10.1089/lap.2013.0418 24494971 PMC4047993

[9] Hiroshima Y, Maawy A, Sato S, Murakami T, Uehara F, Miwa S, et al Hand-held high-resolution fluorescence imaging system for fluorescence-guided surgery of patient and cell-line pancreatic tumors growing orthotopically in nude mice. J Surg Res. 2014;187: 510–517. 10.1016/j.jss.2013.11.1083 24373959 PMC3959586

[10] Kaushal S, McElroy MK, Luiken GA, Talamini MA, Moossa AR, Hoffman RM, et al Fluorophore-conjugated anti-CEA antibody for the intraoperative imaging of pancreatic and colorectal cancer. J Gastrointest Surg. 2008;12: 1938–1950. 10.1007/s11605-008-0581-0 18665430 PMC4396596

[11] Maawy AA, Hiroshima Y, Kaushal S, Luiken GA, Hoffman RM, Bouvet M. Comparison of a chimeric anti-carcinoembryonic antigen antibody conjugated with visible or near-infrared fluorescent dyes for imaging pancreatic cancer in orthotopic nude mouse models. J Biomed Opt. 2013;18: 126016 10.1117/1.JBO.18.12.126016 24356647 PMC3868446

[12] Maawy AA, Hiroshima Y, Zhang Y, Luiken GA, Hoffman RM, Bouvet M. Polyethylene glycol (PEG) linked to near infrared (NIR) dyes conjugated to chimeric anti-carcinoembryonic antigen (CEA) antibody enhances imaging of liver metastases in a nude-mouse model of human colon cancer. PLoS One. 2014;9: e97965 10.1371/journal.pone.0097965 24859320 PMC4032229

[13] Metildi CA, Kaushal S, Lee C, Hardamon CR, Snyder CS, Luiken GA, et al An LED light source and novel fluorophore combinations improve fluorescence laparoscopic detection of metastatic pancreatic cancer in orthotopic mouse models. J Am Coll Surg. 2012;214: 997–1007 e1002. 10.1016/j.jamcollsurg.2012.02.009 22542065 PMC3360832

[14] Metildi CA, Kaushal S, Luiken GA, Hoffman RM, Bouvet M. Advantages of fluorescence-guided laparoscopic surgery of pancreatic cancer labeled with fluorescent anti-carcinoembryonic antigen antibodies in an orthotopic mouse model. J Am Coll Surg. 2014;219: 132–141. 10.1016/j.jamcollsurg.2014.02.021 24768506 PMC4065820

[15] Metildi CA, Kaushal S, Luiken GA, Talamini MA, Hoffman RM, Bouvet M. Fluorescently labeled chimeric anti-CEA antibody improves detection and resection of human colon cancer in a patient-derived orthotopic xenograft (PDOX) nude mouse model. J Surg Oncol. 2014;109: 451–458. 10.1002/jso.23507 24249594 PMC3962702

[16] Metildi CA, Kaushal S, Pu M, Messer KA, Luiken GA, Moossa AR, et al Fluorescence-guided surgery with a fluorophore-conjugated antibody to carcinoembryonic antigen (CEA), that highlights the tumor, improves surgical resection and increases survival in orthotopic mouse models of human pancreatic cancer. Ann Surg Oncol. 2014;21: 1405–1411. 10.1245/s10434-014-3495-y 24499827 PMC4334378

[17] Tran Cao HS, Kaushal S, Metildi CA, Menen RS, Lee C, Snyder CS, et al Tumor-specific fluorescence antibody imaging enables accurate staging laparoscopy in an orthotopic model of pancreatic cancer. Hepatogastroenterology. 2012;59: 1994–1999. 10.5754/hge11836 22369743 PMC4096574

[18] Bouvet M, Wang J, Nardin SR, Nassirpour R, Yang M, Baranov E, et al Real-time optical imaging of primary tumor growth and multiple metastatic events in a pancreatic cancer orthotopic model. Cancer Res. 2002;62: 1534–1540. 11888932

[19] Bouvet M, Yang M, Nardin S, Wang X, Jiang P, Baranov E, et al Chronologically-specific metastatic targeting of human pancreatic tumors in orthotopic models. Clin Exp Metastasis. 2000;18: 213–218. 11315094 10.1023/a:1006767405609

[20] Fu X, Guadagni F, Hoffman RM. A metastatic nude-mouse model of human pancreatic cancer constructed orthotopically with histologically intact patient specimens. Proc Natl Acad Sci U S A. 1992;89: 5645–5649. 1608975 10.1073/pnas.89.12.5645 PMC49349

[21] Furukawa T, Kubota T, Watanabe M, Kitajima M, Hoffman RM. A novel "patient-like" treatment model of human pancreatic cancer constructed using orthotopic transplantation of histologically intact human tumor tissue in nude mice. Cancer Res. 1993;53: 3070–3072. 8319214

[22] Yamauchi K, Yang M, Jiang P, Xu M, Yamamoto N, Tsuchiya H, et al Development of real-time subcellular dynamic multicolor imaging of cancer-cell trafficking in live mice with a variable-magnification whole-mouse imaging system. Cancer Res. 2006;66: 4208–4214. 16618743 10.1158/0008-5472.CAN-05-3927

[23] Nakajima T, Sano K, Choyke PL, Kobayashi H. Improving the efficacy of Photoimmunotherapy (PIT) using a cocktail of antibody conjugates in a multiple antigen tumor model. Theranostics. 2013;3: 357–365. 10.7150/thno.5908 23781283 PMC3677407

